# Membrane-based cancer nanovaccines: the time is now

**DOI:** 10.1093/qjmed/hcad089

**Published:** 2023-05-17

**Authors:** G Zhao, Y Jiang, P Ma, S Wang, G Nie, N Li

**Affiliations:** Clinical Trial Center, National Cancer Center/National Clinical Research Center for Cancer/Cancer Hospital, Chinese Academy of Medical Sciences and Peking Union Medical College, Beijing 100021, China; Clinical Trial Center, National Cancer Center/National Clinical Research Center for Cancer/Cancer Hospital, Chinese Academy of Medical Sciences and Peking Union Medical College, Beijing 100021, China; Clinical Trial Center, National Cancer Center/National Clinical Research Center for Cancer/Cancer Hospital, Chinese Academy of Medical Sciences and Peking Union Medical College, Beijing 100021, China; Clinical Trial Center, National Cancer Center/National Clinical Research Center for Cancer/Cancer Hospital, Chinese Academy of Medical Sciences and Peking Union Medical College, Beijing 100021, China; CAS Key Laboratory for Biomedical Effects of Nanomaterials and Nanosafety & CAS Center for Excellence in Nanoscience, National Center for Nanoscience and Technology of China, Zhongguancun, Beijing, China; Clinical Trial Center, National Cancer Center/National Clinical Research Center for Cancer/Cancer Hospital, Chinese Academy of Medical Sciences and Peking Union Medical College, Beijing 100021, China

The burgeoning field of nanovaccines presents the promise of breakthrough developments in cancer treatment.^1^ A nanoscale cancer vaccine platform can generally be formulated by co-encapsulating tumor antigens and immune adjuvant(s) into a suitable carrier to elicit antigen-specific immune responses, leading to safe and potent antitumor immunity.^2^ Taking advantage of cell membranes, as crucial mediators of cell–cell interactions and intercellular communication, numerous biomimetic, cell membrane-camouflaged nanovaccines have been developed to enhance the receptor–ligand interactions, and elicit robust antitumor immune responses.^3^ Given the growing interest in this approach, here, we present a systematic review of the progress of membrane-based cancer nanovaccine application in both pre-clinical and clinical studies published until March 2023. We also highlight key trends and discuss emerging, novel membrane-based cancer nanovaccines, including hybrid membrane nanovaccines, microcapsule nanovaccines and exosome membrane-based nanovaccines. Finally, we provide our outlook on this technique to guide future research in this field.

## The membrane-based cancer nanovaccine R&D landscape

### Vaccine platform diversity

With regards to membrane selection, 34/125 (27%) nanovaccines in development use single cancer cell membrane formulations (Supplementary Figure S1), which possess unique homotypic targeting and immune escape properties. Nanovaccines developed based on other popular membrane types include 19 (15%) utilizing immune cell membranes and another 19 (15%) that utilize bacteria-derived outer membrane vesicles, both of which also exhibit tumor targeting and immune stimulation capacity. For membrane formulation, 42 (34%) membrane-derived nanovaccines developed so far are made of innovative formulations of hybrid membranes (27, 22%), exosomes (13, 10%) and microcapsules (2, 2%). Meanwhile, multiple materials have been used to comprise the core in different biomimetic nanovaccines, including polymer-based nanoparticles, gold, tumor antigens and chemotherapy agents, among others. As for synthetic inner cores, 40 available nanovaccines have utilized polymeric organic materials due to their excellent biodegradability and minimal adverse effects; poly (lactic-co-glycolic acid) (PLGA)^4^ approved by the US Food and Drug Administration (US FDA) is the most widely used polymeric material (Supplementary Figure S1). In addition to polymeric organic materials, inorganic materials, tumor antigens/functional peptides and small molecule inhibitors have been fabricated into the inner core for 32, 11 and 17 membrane-derived nanovaccines, respectively.

### Adjuvant choice and combination strategy

Immunostimulatory vaccine adjuvants play a crucial role in facilitating strong antitumor immune responses. Most scientists choose toll-like receptor (TLR) agonists (e.g. CpG oligonucleotides, MPLA, R837, R848, etc.) as immunoadjuvants to potentiate immune responses. Thus far, 50 nanovaccines have been developed together with immune adjuvants, among which TLR agonists were the dominant adjuvant type (32; 64%), while stimulator of interferon genes (STING) agonists (3), and other immune adjuvants (15) accounted for the remaining 36% of adjuvants used (Supplementary Figure S1). Combination treatment is another method to enhance vaccination efficiency and achieve longer-lasting tumor regression. Immune checkpoint blockade (ICB), photothermal therapy (PTT) and photodynamic therapy (PDT) are the three most common strategies used in combination with nanovaccines, all of which have exhibited huge potential for clinical application. Anti-PD1 treatment is the most popular ICB used in combination with membrane-based nanovaccines, accounting for 22% of all combination strategies, while PTT and PDT account for 22% and 16% of all combination strategies, respectively.

### Profile of vaccine developers

Among the available membrane-based nanovaccine candidates, 122 (98%) are developed by academic institutes or university affiliated hospitals, while only 3 (2%) are promoted by pharmaceutical industries from the USA and France (Supplementary Figure S1). Most (87, 62%) of membrane-based nanovaccine developers are from China, followed by the USA (26, 28%), other countries in Asia except China (8, 7%) and Europe (4, 3%) (Supplementary Figure S1). The leading developers currently have 125 membrane-based vaccine candidates in their respective pipelines, among which 112 (90%) are under preclinical studies (Supplementary Table S1) and 13 (10%) enter the clinical trial phase (Supplementary Table S2).

### Frontiers in membrane-based vaccine development

**Figure 1.**
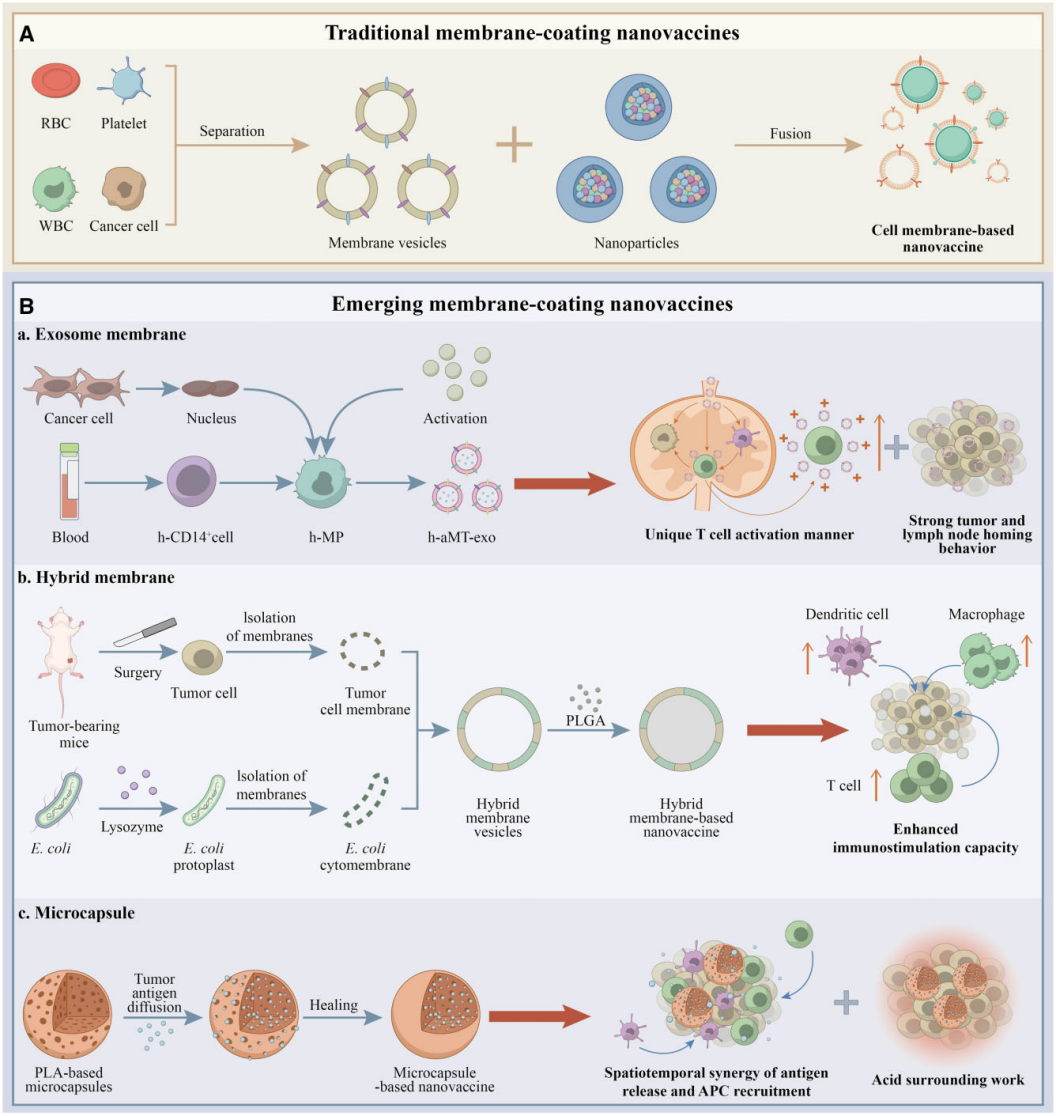
Schematic diagram of traditional and emerging membrane-based cancer nanovaccine fabrication strategies. (**A**) Traditional membrane-based cancer nanovaccines. A single-cell type (e.g. erythrocytes, lymphocytes, etc.) are collected as appropriate. Next, the cell membranes are separated via freeze–thaw cycles and hypotonic treatment for anucleate cells and sonication, nitrogen cavitation or mechanical homogenization for nucleated cells. Finally, the core materials are coated with the membranes, generating the membrane-based cancer nanovaccines. (**B**) Emerging membrane-based cancer nanovaccines. (**a**) Nuclei isolated from tumor cells are introduced into activated M1-like macrophages to form chimeric exosomes (aMT-exos), which can then enter lymph nodes to prime T-cell activation in a ‘direct exosome interaction’ manner and generate vesicles with a strong tumor homing behavior; **(b)** an adjuvant and antigen co-delivery nanovaccine based on *Escherichia coli* cytoplasmic membranes and tumor cell membranes from resected autologous tumor tissues elicited a strong immune stimulation in the tumor microenvironment to enhance tumor killing; **(c)** a novel microcapsule-based vaccine based on the unique self-healing feature of polylactic acid materials can generate a favorable immunization microenvironment in tumor sites together with effective tumor antigen release and immune cell recruitment.

The unique features of biomimetic nanovaccines have attracted increasing attention from researchers and the pharmaceutical industry. Here, we have highlighted some advancements in the development of membrane-based cancer nanovaccines, such as the use of hybrid membranes, exosomes and microcapsules, all of which confer unique and outstanding antitumor capacities to their respective formulations (Figure 1).

The membrane fraction of hybrid cell membrane-derived nanovaccines can be derived from different cell types, including cancer cells, dendritic cells and erythrocytes.^5^ Hybrid membranes imbue nanoparticles with novel characteristics unavailable in other cell membrane-based NPs, such as enhanced antigen delivery efficiency and precise targeting via lymph node guiding.^5^ There are currently 27 studies describing hybrid membrane vaccine development, in which nearly half (12) are being evaluated in clinical trials. This high rate of clinical translation reflects the antitumor potential of the hybrid membrane approach. Nie *et al*. developed an adjuvant and antigen co-delivery nanovaccine based on *Escherichia coli* outer membrane vesicles and tumor cell membranes from surgically resected tumor tissue. This tumor vaccine effectively induced dendritic cells (DC) maturation and splenic T-cell activation, leading to a potent antitumor effect in various tumor models.^6^ In addition to hybrid membranes, exosomes have also been utilized to fabricate nanovaccines. Exosomes are cell-derived nanovesicles comprising various molecular constituents that are naturally secreted by almost all types of cells, including tumor cells and immune cells.^7^ A pre-clinical investigation demonstrated that exosomes generated from dendritic cells can serve as effective cancer nanovaccines due to their inherited ability to induce antitumor responses.^8^ Following this strategy, Wei *et al*. designed a chimeric-membrane nanovaccine based on exosomes from macrophage-tumor hybrid cells. Their nanovaccine targeted lymph nodes and primed T-cell responses in a unique ‘direct exosome interaction’ manner. This nanovaccine can induce long-lasting tumor regression and improve the survival of various cancer models, especially when combined with anti-PD1 therapy.^9^ As for microcapsule-based nanovaccines, Wang *et al*.^10^ developed a self-healing microcapsule strategy that can generate a favorable tumor microenvironment *in situ*, wherein antigen release, antigen presentation cell recruitment and acid microenvironment work in a synergetic manner to promote tumor regression. Another advantage of this vaccination is that it possesses established safety profile and high translation potential because it is based on FDA-approved polylactic acid. Currently, Wei *et al*. have designed two microcapsule-based nanovaccines, both of which have exhibited potent antitumor activity in various hematological cancer and solid tumor models.

### Government policies to support new vaccine development in China

In order to promote the development of new vaccine platforms in China, the Ministry of Industry and Information Technology and the National Medical Products Administration of China have promulgated a series of policies that support new vaccine development, of which the most relevant are the *Guidelines for pharmaceutical industry development planning* and the *‘14th five-year’ plan*. These documents indicate that the Chinese government supports innovative drug design and investigation on new vaccines to achieve breakthroughs. These specific supportive policies have ignited enthusiasm among Chinese scientists for development of new vaccines, not the least of which include membrane-based personalized vaccines.

Research on membrane-based cancer nanovaccine has opened a new avenue toward affordable and efficient cancer vaccines. It is our hope and expectation that this therapeutic area with rise as a bright star in the treatment of cancer in the near future.

## Supplementary Material

hcad089_Supplementary_Data

## References

[1] Sengupta S. Cancer nanomedicine: lessons for immuno-oncology. Trends Cancer 2017; 3:551–60. 28780932 10.1016/j.trecan.2017.06.006

[2] Zhu G , Zhang F, Ni Q, Niu G, Chen X. Efficient nanovaccine delivery in cancer immunotherapy. ACS Nano 2017; 11:2387–92. 28277646 10.1021/acsnano.7b00978

[3] Fang RH , Gao W, Zhang L. Targeting drugs to tumours using cell membrane-coated nanoparticles. Nat Rev Clin Oncol 2023; 20:33–48. 36307534 10.1038/s41571-022-00699-x

[4] Danhier F , Ansorena E, Silva JM, Coco R, Le Breton A, Préat V. PLGA-based nanoparticles: an overview of biomedical applications. J Control Release 2012; 161:505–22. 22353619 10.1016/j.jconrel.2012.01.043

[5] Liao Y , Zhang Y, Blum NT, Lin J, Huang P. Biomimetic hybrid membrane-based nanoplatforms: synthesis, properties and biomedical applications. Nanoscale Horiz 2020; 5:1293–302. 32608425 10.1039/d0nh00267d

[6] Chen L , Qin H, Zhao R, Zhao X, Lin L, Chen Y, et al Bacterial cytoplasmic membranes synergistically enhance the antitumor activity of autologous cancer vaccines. Sci Transl Med 2021; 13:eabc2816. 10.1126/scitranslmed.abc2816 34233949

[7] Kalluri R , LeBleu VS. The biology, function, and biomedical applications of exosomes. Science 2020; 367:eaau6977. 10.1126/science.aau6977 PMC7717626 32029601

[8] Andre F , Chaput N, Schartz NE, Flament C, Aubert N, Bernard J, et al Exosomes as potent cell-free peptide-based vaccine. I. Dendritic cell-derived exosomes transfer functional MHC class I/peptide complexes to dendritic cells. J Immunol 2004; 172:2126–36. 14764678 10.4049/jimmunol.172.4.2126

[9] Wang S , Li F, Ye T, Wang J, Lyu C, Qing S, et al Macrophage-tumor chimeric exosomes accumulate in lymph node and tumor to activate the immune response and the tumor microenvironment. Sci Transl Med 2021; 13:eabb6981. 34644149 10.1126/scitranslmed.abb6981

[10] Xi X , Ye T, Wang S, Na X, Wang J, Qing S, et al Self-healing microcapsules synergetically modulate immunization microenvironments for potent cancer vaccination. Sci Adv 2020; 6:eaay7735. 32494733 10.1126/sciadv.aay7735 PMC7244316

