# Supplementary material for: Membrane-based cancer nanovaccines: the time is now
Source: QJM. 2023 May 17;116(8):621–4. doi: 10.1093/qjmed/hcad089 (PMC10497184; doi:10.1093/qjmed/hcad089)
Supplement: hcad089_Supplementary_Data [file hcad089_supplementary_data.zip › hcad089_Supplementary_Data/Supplymentary_Tables clean.docx]

**Supplementary Table 1. Summary of membrane-based cancer nanovaccines**

| **Vaccine Name** | **Cancer Type** | **Membrane Origin** | **Immunogenic antigens** | **Surface Modification** | **Immune-adjuvant** | **Core materials** | **Combined treatment** | **Lead organization** | | **References** | | |
| --- | --- | --- | --- | --- | --- | --- | --- | --- | --- | --- | --- | --- |
| **Cell membrane** |  |  |  |  |  |  |  |  | |  | | |
| PLGA/STING@EPBM | Melanoma | B16 OVA melanoma cells | Tumor cell antigens and CBP-12 | 12-mer Clec9a binding peptide | cGAMP | PLGA/STING | Radiotherapy | Zhengzhou University/China | | ^1^ | | |
| TOPSi@AcDEX@CCM | Breast cancer | MDA-MB-231 Cells | Tumor cell antigens | Acetalated dextran | Acetalated Dextran (AcDEX) | Thermally oxidized PSi (TOPSi) | NA | University of Helsinki/Finland | | ^2^ | | |
| A/M/C-MNC | Breast cancer | 4T1 cells | Tumor cell antigens | anti-CD205 and Azide CHO | CpG oligodeoxynucleotide | Fe3O4 magnetic nanoclusters | NA | Beijing Institute of Technology/China | | ^3^ | | |
| IMQ/siR@ATM-NVs | Melanoma | B16-F10 melanoma cells | Tumor cell antigens | anti-CD205 antibody, DHPC, DSPE-PEG-FcBP, Cholesterol | R837 | IL-10 siRNA | NA | Yeungnam University/Republic of Korea | | ^4^ | | |
| mEHGZ | Breast cancer | 4T1 cells | Tumor cell antigens | Calreticulin | Cell membrane with overexpressed CRT | ZIF-8, EPI, Gox and hemin | anti-PD-L1 | Sichuan University/China | | ^5^ | | |
| CMV-CpG/Apt | Melanoma | B16 OVA melanoma cells | Tumor cell antigens | chol-CpG-FAM and chol-aptamer | CpG oligodeoxynucleotide | NA | anti-PD-1 | Shanghai Jiao Tong University/China | | ^6^ | | |
| ExtraCRAd | Melanoma and lung cancer | B16 OVA, B16-F10 melanoma cells and LL/2 lung cancer cells | Tumor cell antigens | CpG ligands | Virus | Oncolytic adenovirus serotype 5 | NA | University of Helsinki/Finland | | ^7^ | | |
| CM@CaPyro NGs | Melanoma | B16 OVA melanoma cells | Tumor cell antigens | DOPA, DOPC, DSPE-PEG2000 | Calcium phosphate | Calcium pyrophosphate | NA | Peking University/China | | ^8^ | | |
| NP-R@M-M | Melanoma | B16 OVA melanoma cells | Tumor cell antigens | DSPE-PEG-Man | R837 | PLGA | anti-PD-1 | Soochow University/China | | ^9^ | | |
| AP-NPs | Melanoma | B16-F10 melanoma cells | Tumor cell antigens | Engineered CD80 molecules | CpG oligodeoxynucleotide | NA | NA | University of California San Diego/USA | | ^10^ | | |
| Tumor tissue based TMV vaccines | HNSCC | MOC1 and MOC2 cells | Tumor cell antigens | GPI-B7-1 and GPI-IL-12 | NA | NA | anti-PD-1 | Emory University/USA | | ^11^ | | |
| Tumor tissue based TMV vaccines | Breast cancer | 4T1 cells | Tumor cell antigens | GPI-B7-1 and GPI-IL-12 | NA | NA | anti-CTLA4 | Metaclipse Therapeutics Corporation/USA | | ^12^ | | |
| Lp-KR-CCM-A | Breast cancer | 4T1 cells | Tumor cell antigens | Killer Red protein and lipid adjuvant | MPLA | NA | Photodynamics therapy | Seoul National University/Republic of Korea | | ^13^ | | |
| CCNP | Breast cancer | MDA-MB-435 cells | Tumor cell antigens | NA | MPLA | PLGA | NA | University of California San Diego/USA | | ^14^ | | |
| CCMF-PLGA NPs | Melanoma | B16-F10 melanoma cells | Tumor cell antigens | NA | NA | PLGA | NA | Johns Hopkins University/USA | | ^15^ | | |
| Gel-BPQD-CCNVs | Breast cancer | 4T1 cells | Tumor cell antigens | NA | GM-CSF and LPS | Black phosphorus quantum dot | anti-PD-1 | Sun Yat-Sen University/China | | ^16^ | | |
| TOPSi@AcDEX@B16.OVA nanoparticles | Melanoma | B16 OVA melanoma cells | Tumor cell antigens | NA | Acetalated Dextran (AcDEX) | TOPSi core | anti-CTLA4 | University of Helsinki/Finland | | ^17^ | | |
| APMC | Melanoma | B16-F10 melanoma cells | Tumor cell antigens | NA | CpG oligodeoxynucleotide | Aluminum phosphate nanoparticles | NA | Sichuan University/China | | ^18^ | | |
| F13-PEI/OVA nanovaccine | Breast cancer | 4T1 cells | Tumor cell antigens | NA | CpG oligodeoxynucleotide | F13-PEI and OVA | anti-CTLA4 | Soochow University/China | | ^19^ | | |
| CM@MON@DOX | Breast cancer | 4T1 cells | Tumor cell antigens | NA | NA | Mesoporous organosilica nanoparticles-doxorubicin | anti-PD-L1 | Chinese Academy of Sciences/China | | ^20^ | | |
| CCMP@R837 | Breast cancer | 4T1 cells | Tumor cell antigens | NA | R837 | PLGA | NA | Nanjing University of Chinese Medicine/China | | ^21^ | | |
| R@P-IM | Breast cancer | Luc-4T1 cells | Calcinetin and tumor cell antigens | NA | R837 | PLGA | NA | Southwest Jiaotong University/China | | ^22^ | | |
| CCM@(PSiNPs@Au) | Breast cancer | 4T1 cells | Tumor cell antigens | NA | NA | PSiNPs@Au | Photothermal therapy | Nanjing Forestry University/China | | ^23^ | | |
| MPDA-R848@CM | Breast cancer | Surgical tumor-derived cell membranes | Tumor cell antigens | NA | R848 | Mesoporous polydopamine | anti-PD-L1 and Photothermal therapy | Sun Yat-Sen University/China | | ^24^ | | |
| Nanocomposite hydrogel-gel-based individualized vaccine | Breast cancer | 4T1 cells | Tumor cell antigens | Nanocomposite hydrogel-gel | R837 | PLGA and OVA | anti-PD-1 | Soochow University/China | | ^25^ | | |
| PEG-NPs | Melanoma | B16 OVA melanoma cells | Tumor cell antigens | PEG | CpG oligodeoxynucleotide | NA | anti-PD-1 | University of Michigan/USA | | ^26^ | | |
| gCM-MNs | Breast cancer and melanoma | B16-F10 melanoma cells and 4T1 cells | Tumor cell antigens | SIRPα | NA | Magnetic nanoparticles | NA | National Institutes of Health (NIH)/USA | | ^27^ | | |
| S-CM-HPAD | Melanoma | B16-F10 melanoma cells and 4T1 cells | Tumor cell antigens | S-layer protein | NA | DOX-Loaded polymer | Chemotherapy | Zhejiang University/China | | ^28^ | | |
| CpG-CCNPs | Melanoma | B16-F10 melanoma cells | Tumor cell antigens | NA | CpG oligodeoxynucleotide | PLGA | anti-CTLA4 and anti-PD1 | University of California San Diego/USA | | ^29^ | | |
| AMCNP | Acute myeloid leukemia | AML cells | Tumor cell antigens | NA | CpG oligodeoxynucleotide | PLGA | NA | University of California San Diego/USA | | ^30^ | | |
| BCNCCM | Breast cancer | 4T1 cells | Tumor cell antigens | NA | CpG oligodeoxynucleotide | BP-Au-CpG/NGL919 | Photothermal therapy | Guangzhou University of Chinese Medicine/China | | ^31^ | | |
| DBE@CCNPs | Melanoma | B16-F10 melanoma cells | Tumor cell antigens | NA | CpG oligodeoxynucleotide | PEI25k | anti-PD-L1 | Yantai University/China | | ^32^ | | |
| PTh/MnO2 @M | Melanoma | B16 melanoma cells | Tumor cell antigens | NA | NA | PTh/MnO2 | Photodynamics therapy | Wuhan University/China. | | ^33^ | | |
| MSNs@cGAMP@CM-SN21 | Melanoma | B16-F10 melanoma cells | Tumor cell antigens and macropinocytosis-inducing peptide SN21 | NA | cGAMP | Diselenide-bridged mesoporous silica nanoparticles (MSNs) | anti-PD-1 | Chinese Academy of Sciences/China | | ^34^ | | |
| Cancer cell membrane-coated cancer vaccine | Melanoma | B16-F10 melanoma cells | Tumor cell antigens | NA | CpG oligodeoxynucleotide | Mesoporous silica nanoparticles | anti-CTLA4 | Columbia University (New York)/USA | | ^35^ | | |
| M-NP-Ag | Melanoma | B16-F10 melanoma cells | Tumor cell antigens | NA | NA | PLGA, Neoantigens | anti-PD-1 | Chinese Academy of Sciences/China | | ^36^ | | |
| M@HLPC | Glioma | Surgical tumor-derived cell membranes | Tumor cell antigens | NA | NA | oxygen-carrying Hb-O2, LOX, CPPO, and Ce6 | PDT | Shenzhen University/China | | ^37^ | | |
| ASPIRE | Melanoma and Lung cancer | DC | Modified antigens | anti-PD1 antibody and B7-1/2 | AlumOH | NA | NA | Xiamen University/China | | ^38^ | | |
| mini DC | Ovarian cancer | DC | Surface proteins | NA | IL-2 | PLGA+IL-2 | NA | Shanghai Jiao Tong University/China | | ^39^ | | |
| mD@cSMNs | Liver cancer | DC | Surface proteins | NA | NA | SiPCCl2 -hybridized mesoporous silica with coordination of Fe(III)-captopril | PDT | Fujian Medical University/China | | ^40^ | | |
| NanoDCs | Melanoma and Colorectal Tumor | DC | Escherichia coli and B16-OVA or CT26 tumor cells | NA | NA | Ferrous ion self-assembled nanostructures | NA | Chinese Academy of Sciences/China | | ^41^ | | |
| BNs | Melanoma | DC | Tumor cell antigens | NA | NA | PLGA | anti-PD-1 | Shanghai Jiao Tong University/China | | ^42^ | | |
| DC-MVs | Uncertain cancer | DC | Surface proteins | PEG | Monophosphoryl lipid A (MPLA) | SIINFEKL peptide | NA | University of Michigan/USA | | ^43^ | | |
| Pa-M/Ti-NCs | Melanoma and melanoma | Macrophage | Surface proteins | anti-PD-1 antibody | NA | Fe3O4 magnetic nanocluster and TGF-β inhibitor | NA | Beijing Institute of Technology/China | | ^44^ | | |
| GCMNPs | Acute myeloid leukemia and Colorectal Tumor | Leucocytes | Surface proteins | NA | NA | Glycyrrhetinic acid/PLGA | anti-PD-1+Chemotherapy | Jilin University/China | | ^45^ | | |
| NPR@TAMM | Breast cancer | Macrophage | TAM-specific surface antigens | CSF1R | NA | RB-loaded UCNP | PDT | Jilin University/China | | ^46^ | | |
| (C/I)BP@B-A(D)&M1m | Breast cancer and Melanoma | Macrophage | Surface proteins | NA | NA | (C/I)BP@B-A(D) | PDT | Sichuan University/China | | ^47^ | | |
| I-P@NPs@M | Breast cancer | Macrophage | Surface proteins | NA | NA | Ce6/BR-FFVLK-PEG, PTX2-TK and IND-S2 | PDT+Chemotherapy | Sichuan University/China | | ^48^ | | |
| MNGs | Breast cancer | Macrophage | Surface proteins | NA | NA | PEG-PDPA+C14-Gem | anti-PD-L1 | Chinese Academy of Sciences/China | | ^49^ | | |
| D/R/C@SiO2-M | Liver cancer | Macrophage | Surface proteins | NA | R848 | Mesoporous silica nanoparticles+Catalase+Doxorubicin | NA | Southwest Jiaotong University/China | | ^50^ | | |
| PEI-MM-PLGA-DP/OVA | Uncertain cancer | Macrophage | Surface proteins | PEI | Dendrobium devonianum Polygonatum | Dendrobium devonianum Polygonatum+OVA+PLGA | NA | Nanjing University of Chinese Medicine/China | | ^51^ | | |
| MPLP | Breast cancer | Macrophage | Surface proteins | PLGA+ poly I:C+Lecithin+DSPE-PEG-Mal | poly I:C | NA | NA | Chinese Academy of Sciences/China | | ^52^ | | |
| THINR-CXCL10@Gel | Glioma | Macrophage | Tumor cell antigens | NA | NA | Tumor-homing immune nanoregulator+siIDO1 | NA | Shandong University/China | | ^53^ | | |
| NG/BP-PEI-LY | Breast cancer | Neutrophil | Surface proteins | NA | NA | BP-PEI-LY | PTT and PDT | China Pharmaceutical University/China | | ^54^ | | |
| NK-NPs | Breast cancer | NK cells | Surface proteins | NA | NA | TCPP+mPEG-PLGA | PDT | Chinese Academy of Sciences/China | | ^55^ | | |
| TCMNPs | Melanoma | T cell | Plasma-membrane proteins | PD1, LFA-1, FasL, TGF-Β1r | NA | PLGA | Chemotherapy | Seoul National University/Republic of Korea | | ^56^ | | |
| BPQD-RMNV | Breast cancer | Erythrocytes | Surface proteins | NA | NA | Black phosphorus quantum dot | PTT and anti-PD-1 | Shenzhen University/China | | ^57^ | | |
| NR P+I | Melanoma | Erythrocytes | Surface proteins | IL-2 | IL-2 | CECm+HTCCm+PTX loaded HP-β-CD-A | Chemotherapy | Huazhong University of Science and Technology/China. | | ^58^ | | |
| Man-RBC-NPhgp | Melanoma | Erythrocytes | Surface proteins | DSPE-PEG-Man | MPLA | PLGA+hgp10025-33 | NA | Huazhong University of Science and Technology/China. | | ^59^ | | |
| Nano-ghost glyconanoparticles | Colorectal Tumor | Erythrocytes | Surface proteins | Neu5Gc antibody | Freud’s complete adjuvant | NA | NA | Tel Aviv University/Israel | | ^60^ | | |
| Blood clot vaccine | Breast cancer and Melanoma | Erythrocytes | Surface proteins | NA | CpG oligodeoxynucleotide | NA | Anti-PD1 | Soochow University/China | | ^61^ | | |
| lCUR-DOX@RBC NPs | Melanoma | Erythrocytes | Surface proteins | NA | LCUR | Doxorubicin + low molecular weight curdlan | Chemotherapy | Jinan University/China | | ^62^ | | |
| PNP-R848 | Breast cancer and Colorectal Tumor | Platelet | Platelet surface proteins | NA | R848 | PLGA | NA | Cello Therapeutics, Inc./USA | | ^63^ | | |
| OMV-LL-Mrna | Melanoma and Colorectal Tumor | E. coli OMVs | OMV immunogens | ClyA protein | Listeriolysin O | NA | NA | Chinese Academy of Sciences/China | | ^64^ | | |
| OMV–Ag–mFc | Melanoma and Colorectal Tumor | E. coli OMVs | Tumor antigens and OMV immunogens | ClyA-Ag-mFc | Poly(I:C) + Adpgk | NA | NA | Chinese Academy of Sciences/China | | ^65^ | | |
| *E. coli ∆msbB* OMVs | Colorectal Tumor | E. coli OMVs | OMV immunogens | NA | NA | NA | NA | Pohang University of Science and Technology/Republic of Korea | | ^66^ | | |
| BFGF-OMV | Melanoma | E. coli OMVs | OMV immunogens | NA | NA | BFGF protein | NA | Peking Union Medical College/China | | ^67^ | | |
| OMV@CaPs | Breast cancer and Colorectal Tumor | E. coli OMVs | OMV immunogens | Calcium phosphate | NA | NA | PTT | Chinese Academy of Sciences/China | | ^68^ | | |
| OMV-PD1 | Melanoma | E. coli OMVs | OMV immunogens | PD-1 | NA | NA | NA | Chinese Academy of Sciences/China | | ^69^ | | |
| Bioengineered bacteria-derived OMVs | Melanoma and Colorectal Tumor | E. coli OMVs | Tumor antigens and OMV immunogens | ClyA protein | Poly(I:C) + Adpgk | NA | NA | Chinese Academy of Sciences/China | | ^70^ | | |
| Au-OMV | Glioblastoma | E. coli OMVs | OMV immunogens | NA | NA | Au | Radiotherapy | Far Eastern Memorial Hospital/China | | ^71^ | | |
| Motor-OMV | Melanoma and Colorectal Tumor | E. coli OMVs | OMV immunogens | NA | NA | Mg, Au, TiO2, PLGA, chitosan | NA | University of California San Diego/USA | | ^72^ | | |
| 1-MT@OMV-Mal | Colorectal Tumor | E. coli OMVs | Tumor antigens and OMV immunogens | Maleimide-PEG4-NHS | OMVs | 1-methyl-tryptophan | PTT | Chinese Academy of Sciences/China | | ^73^ | | |
| DOX/Ce6-OMVs@ M | Triple-negative breast cancer | E. coli OMVs | OMV immunogens | NA | NA | Doxorubicin+Ce6 | Chemotherapy+PDT | Central South University/China | | ^74^ | | |
| PEG/Se@OMV-CD47nb | Colorectal Tumor | E. coli OMVs | OMV immunogens | CD47 nanobody and DBCO-PEG/Se | NA | NA | Radiotherapy | Chinese Academy of Sciences/China | | ^75^ | | |
| FMO NPs | Melanoma | E. coli OMVs | OMV immunogens | NA | NA | Fe3O4-MnO2 | PTT | Binzhou Medical University/China | | ^76^ | | |
| DOX-OMV | Lung cancer | K. pneumonia OMVs | OMV immunogens | NA | NA | Doxorubicin | Chemotherapy | Fudan University/China | | ^77^ | | |
| BNP | Neuroblastoma and melanoma | Mycobacterium smegmatis OMVs | OMV immunogens | Mal-PEG4-NHS | CpG | PC7A/CpG polyplex core | Radiotherapy | University of Wisconsin/USA | | ^78^ | | |
| ORFT nanoparticles | Melanoma | Salmonella OMVs | OMV immunogens | DSPE-PEG-RGD | NA | Tegafur-F127 nanomicelles | NA | Zhejiang University/China | | ^79^ | | |
| S.t ΔpG-OMVs | Colorectal Tumor | Salmonella OMVs | OMV immunogens | NA | NA | NA | PTT | Soochow University/China | | ^80^ | | |
| CAT-Ce6@OMV-aPDL1 | Breast cancer | Salmonella OMVs | OMV immunogens | a PDL1 | NA | CAT-Ce6 | PDT | Jilin University/China | | ^81^ | | |
| siRNA@PLOV | Hepatocellular carcinoma | Salmonella OMVs | Tumor antigens and OMV immunogens | NA | OMVs | NA | Photothermal therapy | China Pharmaceutical University/China | | ^82^ | | |
|  |  |  |  |  |  |  |  |  | |  | | |
| **Hybrid membrane** | |  |  |  |  |  |  |  |  | |  |  |
| PCN@FMs | Breast cancer | 4T1 cancer cells and DC cells | Tumor cell antigens | NA | NA | PCN-224 | PDT | Wuhan University/China. | | ^83^ | | |
| R837@HM-NPs | Breast cancer | 4T1 cancer cells and DC cells | Tumor cell antigens | NA | R837 | Mesoporous silica nanoparticle (MSN) | anti-PD-1 | Tianjin Medical University/China | | ^84^ | | |
| MOF@FM | Breast cancer | 4T1 cancer cells and DC cells | Tumor cell antigens | NA | NA | PCN-224 | NA | Wuhan University/China. | | ^85^ | | |
| DMNPs | Glioma and colon cancer | MC38 cancer cells and DC cells | Tumor cell antigens | NA | CpG oligodeoxynucleotide | CpG oligodeoxynucleotide | NA | Sun Yat-Sen University/China | | ^86^ | | |
| DNS-[C6&DC]m | Glioma | C6 cancer cells and DC cells | Tumor cell antigens | NA | NA | Docetaxel+surfactant SDC | Chemotherapy | Beijing Institute of Pharmacology and Toxicology/China | | ^87^ | | |
| FCM-NPs | Overian cancer | ID8 overian cancer cells and DC cells | Tumor cell antigens | NA | CpG oligodeoxynucleotide | PLGA | NA | Tianjin Medical University/China | | ^88^ | | |
| HM-NPs | Breast cancer and Melanoma | 4T1 cancer cells and OMV | Tumor antigens and OMV immunogens | NA | NA | PLGA | NA | Chinese Academy of Sciences/China | | ^89^ | | |
| m TOMV | Breast cancer | 4T1 cancer cells and OMV | Tumor cell antigens | NA | NA | NA | NA | Wuhan University/China. | | ^90^ | | |
| MGTe | Breast cancer | 4T1 cancer cells and OMV | Tumor antigens and OMV immunogens | NA | NA | Glutathione decorated Te nanoparticles | Raditherapy | Wuhan University/China. | | ^91^ | | |
| PI@EPV | Melanoma | B16 cancer cells and OMV | Tumor antigens and OMV immunogens | NA | NA | Indocyanine green+PLGA | PTT | Zhejiang University/China | | ^92^ | | |
| HPDA@[OMV-CC] | Melanoma | B16 cancer cells and OMV | Tumor antigens and OMV immunogens | NA | NA | HPDA NPs | PTT | Anhui Science and Technology University/China | | ^93^ | | |
| nano-Ag@erythrosomes | Breast cancer and Melanoma | B16 cancer cells and RBC | Tumor cell antigens | NA | NA | NA | anti-PD-L1 | Soochow University/China | | ^94^ | | |
| ICGCQ@RCm NPs | Melanoma | B16 cancer cells and RBC | Tumor cell antigens | NA | NA | Indocyanine green and chloroquine | PTT | Southern Medical University/China | | ^95^ | | |
| Fe3O4-ICG@IRM | Overian cancer | ID8 ovarian cancer cells and RBC | Tumor cell antigens | NA | NA | Indocyanine green-loaded magnetic nanoparticles | PTT | Wuhan University/China. | | ^96^ | | |
| hNVs | Breast cancer and Melanoma | M1 Macrophag+Platelet+Cancer cells | Tumor cell antigens | SIRPα | STING agonist | STING agonist | NA | National Institutes of Health (NIH)/USA | | ^97^ | | |
|  |  |  |  |  |  |  |  |  | |  | | |
| **Exosomes** |  |  |  |  |  |  |  |  | |  | | |
| M1NVs | Colorectal Tumor | M1 Macrophage | Surface proteins | NA | NA | NA | anti-PD-L1 | Seoul National University/Republic of Korea | | ^98^ | | |
| CD8+ T cell-derived extracellular vesicles | Breast cancer | CD8+ T cell | Surface proteins | NA | NA | NA | NA | Mie University/Japan | | ^99^ | | |
| NK cell-derived extracellular vesicles | Melanoma | NK cells | Surface proteins | NA | NA | NA | NA | Kyungpook National University/Republic of Korea | | ^100^ | | |
| Tumor Cell-associated Exosomes | Lung cancer | Tumor cells | Surface proteins | NA | NA | NA | NA | Jinan University/China | | ^101^ | | |
| hEX@BP | Lung cancer | Tumor cells | Surface proteins | NA | NA | Back phosphorus quantum dots | PTT | Jinan University/China | | ^102^ | | |
| HELA-Exos | Breast cancer | Tumor cells | Surface proteins | NA | Hiltonol | Immunogenic cell death inducers | NA | Wuhan University/China. | | ^103^ | | |
| M1OVA-Exos | Melanoma | M1 Macrophage | Surface proteins | NA | NA | NA | NA | Hebei University/China | | ^104^ | | |
| aMT-exos | Lymphoma and breast and melanoma | Macrophage-tumor hybrid cells | Surface proteins | NA | NA | NA | anti-PD-1 | Chinese Academy of Sciences/China | | ^105^ | | |
| γδ-T-Exos | Nasopharyngeal carcinoma | γδ-T cells | Surface proteins | NA | NA | NA | Radiotherapy | University of Hong Kong/China | | ^106^ | | |
| DEXP&A2&N | Hepatocellular carcinoma | DC | Surface proteins | P47-P, AFP212-A2 and N1ND-N | NA | NA | NA | Tianjin Medical University/China | | ^107^ | | |
| MUC1-Dex | Melanoma | DC | Surface proteins | MUC1 | NA | NA | NA | Nankai University/China | | ^108^ | | |
| DC-derived extracellular vesicles | Melanoma | DC | Surface proteins | NA | NA | Neoantigens | NA | Peking University/China | | ^109^ | | |
| CpG- exo /TGM | Glioma | Endogenous serum exosomes | Surface proteins | DSPE-PEG-CpG | CpG oligodeoxynucleotide | Tanshinone IIA and glycyrrhizic acid | NA | Nanjing University of Chinese Medicine/China | | ^110^ | | |
|  |  |  |  |  |  |  |  |  | |  | | |
| **Microcapsules** |  |  |  |  |  |  |  |  | |  | | |
| Self-healing microcapsules | Leukaemia | Degradable poly(lactic acid) microcapsules | NA | NA | NA | Leukaemia-associated epitope peptides and anti-PD1 | anti-PD-1 | Southern Medical University/China | | ^111^ | | |
| Self-healing microcapsules | Lymphoma and melanoma | Degradable poly(lactic acid) microcapsules | NA | NA | NA | Tumor antigens | NA | Chinese Academy of Sciences/China | | ^112^ | | |

**References**

1 Gou, S. *et al.* Engineered Nanovaccine Targeting Clec9a(+) Dendritic Cells Remarkably Enhances the Cancer Immunotherapy Effects of STING Agonist. *Nano Lett* **21**, 9939-9950, doi:10.1021/acs.nanolett.1c03243 (2021).

2 Fontana, F. *et al.* Multistaged Nanovaccines Based on Porous Silicon@Acetalated Dextran@Cancer Cell Membrane for Cancer Immunotherapy. *Adv Mater* **29**, doi:10.1002/adma.201603239 (2017).

3 Li, F. *et al.* Engineering Magnetosomes for High-Performance Cancer Vaccination. *ACS Cent Sci* **5**, 796-807, doi:10.1021/acscentsci.9b00060 (2019).

4 Phung, C. D. *et al.* Nanovaccines silencing IL-10 production at priming phase for boosting immune responses to melanoma. *J. Control. Release* **338**, 211-223, doi:10.1016/j.jconrel.2021.08.031 (2021).

5 Li, Z. *et al.* A tumor cell membrane-coated self-amplified nanosystem as a nanovaccine to boost the therapeutic effect of anti-PD-L1 antibody. *Bioact Mater* **21**, 299-312, doi:10.1016/j.bioactmat.2022.08.028 (2023).

6 Liu, B. *et al.* Equipping Cancer Cell Membrane Vesicles with Functional DNA as a Targeted Vaccine for Cancer Immunotherapy. *Nano Lett* **21**, 9410-9418, doi:10.1021/acs.nanolett.1c02582 (2021).

7 Fusciello, M. *et al.* Artificially cloaked viral nanovaccine for cancer immunotherapy. *Nat Commun* **10**, 5747, doi:10.1038/s41467-019-13744-8 (2019).

8 Li, M. *et al.* A biomimetic antitumor nanovaccine based on biocompatible calcium pyrophosphate and tumor cell membrane antigens. *Asian J Pharm Sci* **16**, 97-109, doi:10.1016/j.ajps.2020.06.006 (2021).

9 Yang, R. *et al.* Cancer Cell Membrane-Coated Adjuvant Nanoparticles with Mannose Modification for Effective Anticancer Vaccination. *ACS Nano* **12**, 5121-5129, doi:10.1021/acsnano.7b09041 (2018).

10 Jiang, Y. *et al.* Engineered Cell-Membrane-Coated Nanoparticles Directly Present Tumor Antigens to Promote Anticancer Immunity. *Adv Mater* **32**, e2001808, doi:10.1002/adma.202001808 (2020).

11 Bommireddy, R. *et al.* Tumor Membrane Vesicle Vaccine Augments the Efficacy of Anti-PD1 Antibody in Immune Checkpoint Inhibitor-Resistant Squamous Cell Carcinoma Models of Head and Neck Cancer. *Vaccines (Basel)* **8**, doi:10.3390/vaccines8020182 (2020).

12 Pack, C. D. *et al.* Tumor membrane-based vaccine immunotherapy in combination with anti-CTLA-4 antibody confers protection against immune checkpoint resistant murine triple-negative breast cancer. *Hum. Vaccin. Immunother.* **16**, 3184-3193, doi:10.1080/21645515.2020.1754691 (2020).

13 Kim, H. Y. *et al.* Immunomodulatory Lipocomplex Functionalized with Photosensitizer-Embedded Cancer Cell Membrane Inhibits Tumor Growth and Metastasis. *Nano Lett* **19**, 5185-5193, doi:10.1021/acs.nanolett.9b01571 (2019).

14 Fang, R. H. *et al.* Cancer cell membrane-coated nanoparticles for anticancer vaccination and drug delivery. *Nano Lett* **14**, 2181-2188, doi:10.1021/nl500618u (2014).

15 Jin, J. *et al.* Human Cancer Cell Membrane-Coated Biomimetic Nanoparticles Reduce Fibroblast-Mediated Invasion and Metastasis and Induce T-Cells. *ACS Appl Mater Interfaces* **11**, 7850-7861, doi:10.1021/acsami.8b22309 (2019).

16 Ye, X. *et al.* Surgical Tumor-Derived Personalized Photothermal Vaccine Formulation for Cancer Immunotherapy. *ACS Nano* **13**, 2956-2968, doi:10.1021/acsnano.8b07371 (2019).

17 Fontana, F. *et al.* Biohybrid Vaccines for Improved Treatment of Aggressive Melanoma with Checkpoint Inhibitor. *ACS Nano* **13**, 6477-6490, doi:10.1021/acsnano.8b09613 (2019).

18 Gan, J. *et al.* Tumor cell membrane enveloped aluminum phosphate nanoparticles for enhanced cancer vaccination. *J. Control. Release* **326**, 297-309, doi:10.1016/j.jconrel.2020.07.008 (2020).

19 Xu, J. *et al.* A general strategy towards personalized nanovaccines based on fluoropolymers for post-surgical cancer immunotherapy. *Nat Nanotechnol* **15**, 1043-1052, doi:10.1038/s41565-020-00781-4 (2020).

20 Shao, D. *et al.* Biomimetic Diselenide-Bridged Mesoporous Organosilica Nanoparticles as an X-ray-Responsive Biodegradable Carrier for Chemo-Immunotherapy. *Adv Mater* **32**, e2004385, doi:10.1002/adma.202004385 (2020).

21 Xiao, L. *et al.* Biomimetic cytomembrane nanovaccines prevent breast cancer development in the long term. *Nanoscale* **13**, 3594-3601, doi:10.1039/d0nr08978h (2021).

22 Xiong, X. *et al.* Personalized Nanovaccine Coated with Calcinetin-Expressed Cancer Cell Membrane Antigen for Cancer Immunotherapy. *Nano Lett* **21**, 8418-8425, doi:10.1021/acs.nanolett.1c03004 (2021).

23 Li, J. *et al.* Multifunctional Biomimetic Nanovaccines Based on Photothermal and Weak-Immunostimulatory Nanoparticulate Cores for the Immunotherapy of Solid Tumors. *Adv Mater* **34**, e2108012, doi:10.1002/adma.202108012 (2022).

24 Li, T. *et al.* Surgical Tumor-Derived Photothermal Nanovaccine for Personalized Cancer Therapy and Prevention. *Nano Lett* **22**, 3095-3103, doi:10.1021/acs.nanolett.2c00500 (2022).

25 Meng, Z. *et al.* Ultrasound-Mediated Remotely Controlled Nanovaccine Delivery for Tumor Vaccination and Individualized Cancer Immunotherapy. *Nano Lett* **21**, 1228-1237, doi:10.1021/acs.nanolett.0c03646 (2021).

26 Ochyl, L. J. *et al.* PEGylated tumor cell membrane vesicles as a new vaccine platform for cancer immunotherapy. *Biomaterials* **182**, 157-166, doi:10.1016/j.biomaterials.2018.08.016 (2018).

27 Rao, L. *et al.* Activating Macrophage-Mediated Cancer Immunotherapy by Genetically Edited Nanoparticles. *Adv Mater* **32**, e2004853, doi:10.1002/adma.202004853 (2020).

28 Wu, M. *et al.* Surface-Layer Protein-Enhanced Immunotherapy Based on Cell Membrane-Coated Nanoparticles for the Effective Inhibition of Tumor Growth and Metastasis. *ACS Appl Mater Interfaces* **11**, 9850-9859, doi:10.1021/acsami.9b00294 (2019).

29 Kroll, A. V. *et al.* Nanoparticulate Delivery of Cancer Cell Membrane Elicits Multiantigenic Antitumor Immunity. *Adv Mater* **29**, doi:10.1002/adma.201703969 (2017).

30 Johnson, D. T. *et al.* Acute myeloid leukemia cell membrane-coated nanoparticles for cancer vaccination immunotherapy. *Leukemia* **36**, 994-1005, doi:10.1038/s41375-021-01432-w (2022).

31 Huang, D. *et al.* In situ photothermal nano-vaccine based on tumor cell membrane-coated black phosphorus-Au for photo-immunotherapy of metastatic breast tumors. *Biomaterials* **289**, 121808, doi:10.1016/j.biomaterials.2022.121808 (2022).

32 Liu, S. *et al.* CD47KO/CRT dual-bioengineered cell membrane-coated nanovaccine combined with anti-PD-L1 antibody for boosting tumor immunotherapy. *Bioact Mater* **22**, 211-224, doi:10.1016/j.bioactmat.2022.09.017 (2023).

33 Li, W., Fan, J. X., Zheng, D. W. & Zhang, X. Z. Tumor Antigen Loaded Nanovaccine Induced NIR-Activated Inflammation for Enhanced Antigen Presentation During Immunotherapy of Tumors. *Small* **18**, e2205193, doi:10.1002/smll.202205193 (2022).

34 Yang, C. *et al.* Biomimetic Nanovaccines Potentiating Dendritic Cell Internalization via CXCR4-Mediated Macropinocytosis. *Adv Healthc Mater* **12**, e2202064, doi:10.1002/adhm.202202064 (2023).

35 Hu, H. *et al.* A Versatile and Robust Platform for the Scalable Manufacture of Biomimetic Nanovaccines. *Adv Sci (Weinh)* **8**, 2002020, doi:10.1002/advs.202002020 (2021).

36 Xiao, P. *et al.* Nanovaccine-Mediated Cell Selective Delivery of Neoantigens Potentiating Adoptive Dendritic Cell Transfer for Personalized Immunization. *Advanced Functional Materials* **28**, 2104068, doi: 10.1002/adfm.202104068(2021).

37 Lu, G. *et al.* Engineered biomimetic nanoparticles achieve targeted delivery and efficient metabolism-based synergistic therapy against glioblastoma. *Nat Commun* **13**, 4214, doi:10.1038/s41467-022-31799-y (2022).

38 Liu, C. *et al.* A nanovaccine for antigen self-presentation and immunosuppression reversal as a personalized cancer immunotherapy strategy. *Nat Nanotechnol* **17**, 531-540, doi:10.1038/s41565-022-01098-0 (2022).

39 Cheng, S. *et al.* Artificial Mini Dendritic Cells Boost T Cell-Based Immunotherapy for Ovarian Cancer. *Adv Sci (Weinh)* **7**, 1903301, doi:10.1002/advs.201903301 (2020).

40 Wang, Y. *et al.* Remodeling Tumor-Associated Neutrophils to Enhance Dendritic Cell-Based HCC Neoantigen Nano-Vaccine Efficiency. *Adv Sci (Weinh)* **9**, e2105631, doi:10.1002/advs.202105631 (2022).

41 Zhang, J. *et al.* Direct Presentation of Tumor-Associated Antigens to Induce Adaptive Immunity by Personalized Dendritic Cell-Mimicking Nanovaccines. *Adv Mater* **34**, e2205950, doi:10.1002/adma.202205950 (2022).

42 Chen, F., Geng, Z., Wang, L., Zhou, Y. & Liu, J. Biomimetic Nanoparticles Enabled by Cascade Cell Membrane Coating for Direct Cross-Priming of T Cells. *Small* **18**, e2104402, doi:10.1002/smll.202104402 (2022).

43 Ochyl, L. J. & Moon, J. J. Dendritic Cell Membrane Vesicles for Activation and Maintenance of Antigen-Specific T Cells. *Adv Healthc Mater* **8**, e1801091, doi:10.1002/adhm.201801091 (2019).

44 Zhang, F. *et al.* Engineering Magnetosomes for Ferroptosis/Immunomodulation Synergism in Cancer. *ACS Nano* **13**, 5662-5673, doi:10.1021/acsnano.9b00892 (2019).

45 Li, Q. *et al.* Glycyrrhetinic acid nanoparticles combined with ferrotherapy for improved cancer immunotherapy. *Acta Biomater* **144**, 109-120, doi:10.1016/j.actbio.2022.03.030 (2022).

46 Chen, C. *et al.* Tumor-Associated-Macrophage-Membrane-Coated Nanoparticles for Improved Photodynamic Immunotherapy. *Nano Lett* **21**, 5522-5531, doi:10.1021/acs.nanolett.1c00818 (2021).

47 Hu, C. *et al.* Phagocyte-membrane-coated and laser-responsive nanoparticles control primary and metastatic cancer by inducing anti-tumor immunity. *Biomaterials* **255**, 120159, doi:10.1016/j.biomaterials.2020.120159 (2020).

48 Liu, R. *et al.* Macrophage-mimic shape changeable nanomedicine retained in tumor for multimodal therapy of breast cancer. *J. Control. Release* **321**, 589-601, doi:10.1016/j.jconrel.2020.02.043 (2020).

49 Li, J. *et al.* Macrophage Membrane-Coated Nano-Gemcitabine Promotes Lymphocyte Infiltration and Synergizes AntiPD-L1 to Restore the Tumoricidal Function. *ACS Nano* **17**, 322-336, doi:10.1021/acsnano.2c07861 (2023).

50 Wen, X. *et al.* A macrophage membrane-coated mesoporous silica nanoplatform inhibiting adenosine A2AR via in situ oxygen supply for immunotherapy. *J. Control. Release* **353**, 535-548, doi:10.1016/j.jconrel.2022.12.001 (2023).

51 Zhang, Z. *et al.* PEI-modified macrophage cell membrane-coated PLGA nanoparticles encapsulating Dendrobium polysaccharides as a vaccine delivery system for ovalbumin to improve immune responses. *Int J Biol Macromol* **165**, 239-248, doi:10.1016/j.ijbiomac.2020.09.187 (2020).

52 Zhou, H. *et al.* In situ poly I:C released from living cell drug nanocarriers for macrophage-mediated antitumor immunotherapy. *Biomaterials* **269**, 120670, doi:10.1016/j.biomaterials.2021.120670 (2021).

53 Zhang, J. *et al.* Immunostimulant hydrogel for the inhibition of malignant glioma relapse post-resection. *Nat Nanotechnol* **16**, 538-548, doi:10.1038/s41565-020-00843-7 (2021).

54 Su, Y. *et al.* A neutrophil membrane-functionalized black phosphorus riding inflammatory signal for positive feedback and multimode cancer therapy. *Materials Horizons.* **2**, 291-297, doi: 10.1039/c9mh01068h (2020).

55 Deng, G. *et al.* Cell-Membrane Immunotherapy Based on Natural Killer Cell Membrane Coated Nanoparticles for the Effective Inhibition of Primary and Abscopal Tumor Growth. *ACS Nano* **12**, 12096-12108, doi:10.1021/acsnano.8b05292 (2018).

56 Kang, M. *et al.* T-Cell-Mimicking Nanoparticles for Cancer Immunotherapy. *Adv Mater* **32**, e2003368, doi:10.1002/adma.202003368 (2020).

57 Liang, X. *et al.* Photothermal cancer immunotherapy by erythrocyte membrane-coated black phosphorus formulation. *J. Control. Release* **296**, 150-161, doi:10.1016/j.jconrel.2019.01.027 (2019).

58 Song, Q. *et al.* Tumor Microenvironment Responsive Nanogel for the Combinatorial Antitumor Effect of Chemotherapy and Immunotherapy. *Nano Lett* **17**, 6366-6375, doi:10.1021/acs.nanolett.7b03186 (2017).

59 Guo, Y. *et al.* Erythrocyte Membrane-Enveloped Polymeric Nanoparticles as Nanovaccine for Induction of Antitumor Immunity against Melanoma. *ACS Nano* **9**, 6918-6933, doi:10.1021/acsnano.5b01042 (2015).

60 Reuven, E. M. *et al.* Biomimetic Glyconanoparticle Vaccine for Cancer Immunotherapy. *ACS Nano* **13**, 2936-2947, doi:10.1021/acsnano.8b07241 (2019).

61 Fan, Q. *et al.* An implantable blood clot-based immune niche for enhanced cancer vaccination. *Sci Adv* **6**, doi:10.1126/sciadv.abb4639 (2020).

62 Lin, M. *et al.* Cell membrane-camouflaged DOX-loaded beta-glucan nanoparticles for highly efficient cancer immunochemotherapy. *Int J Biol Macromol* **225**, 873-885, doi:10.1016/j.ijbiomac.2022.11.152 (2023).

63 Bahmani, B. *et al.* Intratumoral immunotherapy using platelet-cloaked nanoparticles enhances antitumor immunity in solid tumors. *Nat Commun* **12**, 1999, doi:10.1038/s41467-021-22311-z (2021).

64 Li, Y. *et al.* Rapid Surface Display of mRNA Antigens by Bacteria-Derived Outer Membrane Vesicles for a Personalized Tumor Vaccine. *Adv Mater* **34**, e2109984, doi:10.1002/adma.202109984 (2022).

65 Yue, Y. *et al.* Antigen-bearing outer membrane vesicles as tumour vaccines produced in situ by ingested genetically engineered bacteria. *Nat Biomed Eng* **6**, 898-909, doi:10.1038/s41551-022-00886-2 (2022).

66 Kim, O. Y. *et al.* Bacterial outer membrane vesicles suppress tumor by interferon-gamma-mediated antitumor response. *Nat Commun* **8**, 626, doi:10.1038/s41467-017-00729-8 (2017).

67 Huang, W. *et al.* Modified bacterial outer membrane vesicles induce autoantibodies for tumor therapy. *Acta Biomater* **108**, 300-312, doi:10.1016/j.actbio.2020.03.030 (2020).

68 Qing, S. *et al.* Biomineralized Bacterial Outer Membrane Vesicles Potentiate Safe and Efficient Tumor Microenvironment Reprogramming for Anticancer Therapy. *Adv Mater* **32**, e2002085, doi:10.1002/adma.202002085 (2020).

69 Li, Y. *et al.* Bacterial Outer Membrane Vesicles Presenting Programmed Death 1 for Improved Cancer Immunotherapy via Immune Activation and Checkpoint Inhibition. *ACS Nano* **14**, 16698-16711, doi:10.1021/acsnano.0c03776 (2020).

70 Cheng, K. *et al.* Bioengineered bacteria-derived outer membrane vesicles as a versatile antigen display platform for tumor vaccination via Plug-and-Display technology. *Nat Commun* **12**, 2041, doi:10.1038/s41467-021-22308-8 (2021).

71 Chen, M. H., Liu, T. Y., Chen, Y. C. & Chen, M. H. Combining Augmented Radiotherapy and Immunotherapy through a Nano-Gold and Bacterial Outer-Membrane Vesicle Complex for the Treatment of Glioblastoma. *Nanomaterials (Basel)* **11**, doi:10.3390/nano11071661 (2021).

72 Zhou, J. *et al.* Physical Disruption of Solid Tumors by Immunostimulatory Microrobots Enhances Antitumor Immunity. *Adv Mater* **33**, e2103505, doi:10.1002/adma.202103505 (2021).

73 Li, Y. *et al.* Antigen Capture and Immune Modulation by Bacterial Outer Membrane Vesicles as In Situ Vaccine for Cancer Immunotherapy Post-Photothermal Therapy. *Small* **18**, e2107461, doi:10.1002/smll.202107461 (2022).

74 Li, Y. *et al.* Bacterial outer membrane vesicles-based therapeutic platform eradicates triple-negative breast tumor by combinational photodynamic/chemo-/immunotherapy. *Bioact Mater* **20**, 548-560, doi:10.1016/j.bioactmat.2022.05.037 (2023).

75 Feng, Q. *et al.* Engineered Bacterial Outer Membrane Vesicles as Controllable Two-Way Adaptors to Activate Macrophage Phagocytosis for Improved Tumor Immunotherapy. *Adv Mater* **34**, e2206200, doi:10.1002/adma.202206200 (2022).

76 Liu, X. Z. *et al.* Bioengineered Bacterial Membrane Vesicles with Multifunctional Nanoparticles as a Versatile Platform for Cancer Immunotherapy. *ACS Appl Mater Interfaces* **15**, 3744-3759, doi:10.1021/acsami.2c18244 (2023).

77 Kuerban, K. *et al.* Doxorubicin-loaded bacterial outer-membrane vesicles exert enhanced anti-tumor efficacy in non-small-cell lung cancer. *Acta Pharm Sin B* **10**, 1534-1548, doi:10.1016/j.apsb.2020.02.002 (2020).

78 Patel, R. B. *et al.* Development of an In Situ Cancer Vaccine via Combinational Radiation and Bacterial-Membrane-Coated Nanoparticles. *Adv Mater* **31**, e1902626, doi:10.1002/adma.201902626 (2019).

79 Chen, Q. *et al.* Bioengineering Bacterial Vesicle-Coated Polymeric Nanomedicine for Enhanced Cancer Immunotherapy and Metastasis Prevention. *Nano Lett* **20**, 11-21, doi:10.1021/acs.nanolett.9b02182 (2020).

80 Zhuang, Q. *et al.* Bacteria-derived membrane vesicles to advance targeted photothermal tumor ablation. *Biomaterials* **268**, 120550, doi:10.1016/j.biomaterials.2020.120550 (2021).

81 Zhang, J. *et al.* Self-Assembly Catalase Nanocomplex Conveyed by Bacterial Vesicles for Oxygenated Photodynamic Therapy and Tumor Immunotherapy. *Int J Nanomedicine* **17**, 1971-1985, doi:10.2147/IJN.S353330 (2022).

82 Zhai, Y. *et al.* A cascade targeting strategy based on modified bacterial vesicles for enhancing cancer immunotherapy. *J Nanobiotechnology* **19**, 434, doi:10.1186/s12951-021-01193-9 (2021).

83 Liu, W. L. *et al.* Expandable Immunotherapeutic Nanoplatforms Engineered from Cytomembranes of Hybrid Cells Derived from Cancer and Dendritic Cells. *Adv Mater* **31**, e1900499, doi:10.1002/adma.201900499 (2019).

84 Zhao, P. *et al.* Hybrid Membrane Nanovaccines Combined with Immune Checkpoint Blockade to Enhance Cancer Immunotherapy. *Int J Nanomedicine* **17**, 73-89, doi:10.2147/IJN.S346044 (2022).

85 Liu, W. L. *et al.* Cytomembrane nanovaccines show therapeutic effects by mimicking tumor cells and antigen presenting cells. *Nat Commun* **10**, 3199, doi:10.1038/s41467-019-11157-1 (2019).

86 Ma, J. *et al.* Copresentation of Tumor Antigens and Costimulatory Molecules via Biomimetic Nanoparticles for Effective Cancer Immunotherapy. *Nano Lett* **20**, 4084-4094, doi:10.1021/acs.nanolett.9b05171 (2020).

87 Hao, W. *et al.* Hybrid membrane-coated nanosuspensions for multi-modal anti-glioma therapy via drug and antigen delivery. *J Nanobiotechnology* **19**, 378, doi:10.1186/s12951-021-01110-0 (2021).

88 Zhang, L. *et al.* Development of a Dendritic Cell/Tumor Cell Fusion Cell Membrane Nano-Vaccine for the Treatment of Ovarian Cancer. *Front Immunol* **13**, 828263, doi:10.3389/fimmu.2022.828263 (2022).

89 Chen, L. *et al.* Bacterial cytoplasmic membranes synergistically enhance the antitumor activity of autologous cancer vaccines. *Sci Transl Med* **13**, doi:10.1126/scitranslmed.abc2816 (2021).

90 Zou, M. Z., Li, Z. H., Bai, X. F., Liu, C. J. & Zhang, X. Z. Hybrid Vesicles Based on Autologous Tumor Cell Membrane and Bacterial Outer Membrane To Enhance Innate Immune Response and Personalized Tumor Immunotherapy. *Nano Lett* **21**, 8609-8618, doi:10.1021/acs.nanolett.1c02482 (2021).

91 Pan, P. *et al.* A heterogenic membrane-based biomimetic hybrid nanoplatform for combining radiotherapy and immunotherapy against breast cancer. *Biomaterials* **289**, 121810, doi:10.1016/j.biomaterials.2022.121810 (2022).

92 Chen, Q. *et al.* A Hybrid Eukaryotic-Prokaryotic Nanoplatform with Photothermal Modality for Enhanced Antitumor Vaccination. *Adv Mater* **32**, e1908185, doi:10.1002/adma.201908185 (2020).

93 Wang, D. *et al.* Bacterial Vesicle-Cancer Cell Hybrid Membrane-Coated Nanoparticles for Tumor Specific Immune Activation and Photothermal Therapy. *ACS Appl Mater Interfaces* **12**, 41138-41147, doi:10.1021/acsami.0c13169 (2020).

94 Han, X. *et al.* Red blood cell-derived nanoerythrosome for antigen delivery with enhanced cancer immunotherapy. *Sci Adv* **5**, eaaw6870, doi:10.1126/sciadv.aaw6870 (2019).

95 Huang, P. Y. *et al.* Autophagy-inhibiting biomimetic nanodrugs enhance photothermal therapy and boost antitumor immunity. *Biomater Sci* **10**, 1267-1280, doi:10.1039/d1bm01888d (2022).

96 Xiong, J. *et al.* Cancer-Erythrocyte Hybrid Membrane-Camouflaged Magnetic Nanoparticles with Enhanced Photothermal-Immunotherapy for Ovarian Cancer. *ACS Nano* **15**, 19756-19770, doi:10.1021/acsnano.1c07180 (2021).

97 Rao, L. *et al.* Hybrid cellular membrane nanovesicles amplify macrophage immune responses against cancer recurrence and metastasis. *Nat Commun* **11**, 4909, doi:10.1038/s41467-020-18626-y (2020).

98 Choo, Y. W. *et al.* M1 Macrophage-Derived Nanovesicles Potentiate the Anticancer Efficacy of Immune Checkpoint Inhibitors. *ACS Nano* **12**, 8977-8993, doi:10.1021/acsnano.8b02446 (2018).

99 Seo, N. *et al.* Activated CD8(+) T cell extracellular vesicles prevent tumour progression by targeting of lesional mesenchymal cells. *Nat Commun* **9**, 435, doi:10.1038/s41467-018-02865-1 (2018).

100 Zhu, L. *et al.* Exosomes Derived From Natural Killer Cells Exert Therapeutic Effect in Melanoma. *Theranostics* **7**, 2732-2745, doi:10.7150/thno.18752 (2017).

101 Wang, C. *et al.* Tumor Cell-associated Exosomes Robustly Elicit Anti-tumor Immune Responses through Modulating Dendritic Cell Vaccines in Lung Tumor. *Int J Biol Sci* **16**, 633-643, doi:10.7150/ijbs.38414 (2020).

102 Liu, Q. *et al.* Immunogenic exosome-encapsulated black phosphorus nanoparticles as an effective anticancer photo-nanovaccine. *Nanoscale* **12**, 19939-19952, doi:10.1039/d0nr05953f (2020).

103 Huang, L. *et al.* Engineered exosomes as an in situ DC-primed vaccine to boost antitumor immunity in breast cancer. *Mol Cancer* **21**, 45, doi:10.1186/s12943-022-01515-x (2022).

104 Lv, F. *et al.* Therapeutic exosomal vaccine for enhanced cancer immunotherapy by mediating tumor microenvironment. *iScience* **25**, 103639, doi:10.1016/j.isci.2021.103639 (2022).

105 Wang, S. *et al.* Macrophage-tumor chimeric exosomes accumulate in lymph node and tumor to activate the immune response and the tumor microenvironment. *Sci Transl Med* **13**, eabb6981, doi:10.1126/scitranslmed.abb6981 (2021).

106 Wang, X. *et al.* Exosomes derived from gammadelta-T cells synergize with radiotherapy and preserve antitumor activities against nasopharyngeal carcinoma in immunosuppressive microenvironment. *J Immunother Cancer* **10**, doi:10.1136/jitc-2021-003832 (2022).

107 Zuo, B. *et al.* Universal immunotherapeutic strategy for hepatocellular carcinoma with exosome vaccines that engage adaptive and innate immune responses. *J Hematol Oncol* **15**, 46, doi:10.1186/s13045-022-01266-8 (2022).

108 Zhu, H. *et al.* An efficient and safe MUC1-dendritic cell-derived exosome conjugate vaccine elicits potent cellular and humoral immunity and tumor inhibition in vivo. *Acta Biomater* **138**, 491-504, doi:10.1016/j.actbio.2021.10.041 (2022).

109 Li, J. *et al.* Dendritic cell derived exosomes loaded neoantigens for personalized cancer immunotherapies. *J. Control. Release* **353**, 423-433, doi:10.1016/j.jconrel.2022.11.053 (2023).

110 Cui, J. *et al.* Immune Exosomes Loading Self-Assembled Nanomicelles Traverse the Blood-Brain Barrier for Chemo-immunotherapy against Glioblastoma. *ACS Nano*, doi:10.1021/acsnano.2c10219 (2023).

111 Xie, X. *et al.* Therapeutic vaccination against leukaemia via the sustained release of co-encapsulated anti-PD-1 and a leukaemia-associated antigen. *Nat Biomed Eng* **5**, 414-428, doi:10.1038/s41551-020-00624-6 (2021).

112 Xi, X. *et al.* Self-healing microcapsules synergetically modulate immunization microenvironments for potent cancer vaccination. *Sci Adv* **6**, eaay7735, doi:10.1126/sciadv.aay7735 (2020).

**Supplementary Table 2. The Clinical trials of membrane-based cancer nanovaccines**

| **Vaccine Type** | **Vaccine Name** | **Indication** | **Phase** | **Status** | **Combination Treatment** | **NCT Number** | **Lead Organization** | **Country** |
| --- | --- | --- | --- | --- | --- | --- | --- | --- |
| RBC membrane vaccine | GRASPA® | Acute Lymphoblastic Leukemia | 2 | Completed | Chemtherapy | NCT01523782 | ERYtech Pharma | France |
| Hybrid cell membrane vaccine | DC/AML vaccine | Acute Myelogenous Leukemia | 2 | Active, not recruiting | Anti-PD1 | NCT01096602 | Beth Israel Deaconess Medical Center | USA |
| Hybrid cell membrane vaccine | DC/AML vaccine | Acute Myelogenous Leukemia | 1 | Recruiting | Single agent | NCT03679650 | Beth Israel Deaconess Medical Center | USA |
| Hybrid cell membrane vaccine | DC/Tumor Fusion Vaccine | Breast Cancer | 1, 2 | Terminated | Interleukin-12 | NCT00622401 | Beth Israel Deaconess Medical Center | USA |
| Hybrid cell membrane vaccine | DC/Tumor Fusion Vaccine | Glioblastoma | 1, 2 | Recruiting | Interleukin-12, Radiation and Temozolomide | NCT04388033 | Zhejiang University | China |
| Hybrid cell membrane vaccine | DC/Tumor Fusion Vaccine | Melanoma | 1, 2 | Completed | Single agent | NCT00626860 | Beth Israel Deaconess Medical Center | USA |
| Hybrid cell membrane vaccine | DC/MM vaccine | Multiple Myeloma | 2 | Active, not recruiting | Anti-PD1 | NCT01067287 | Beth Israel Deaconess Medical Center | USA |
| Hybrid cell membrane vaccine | DC/Tumor Fusion Vaccine | Multiple Myeloma | 1 | Completed | Single agent | NCT00459069 | Beth Israel Deaconess Medical Center | USA |
| Hybrid cell membrane vaccine | DC/MM vaccine | Multiple Myeloma | 2 | Completed | GM-CSF | NCT03782064 | Beth Israel Deaconess Medical Center | USA |
| Hybrid cell membrane vaccine | DC/MM vaccine | Multiple Myeloma | 2 | Completed | GM-CSF, Melphalan, Lenalidomide | NCT02728102 | University of California, San Francisco | USA |
| Hybrid cell membrane vaccine | DC/Tumor Fusion Vaccine | Ovarian Cancer | 2 | Active, not recruiting | GM-CSF | NCT00799110 | Massachusetts General Hospital | USA |
| Hybrid cell membrane vaccine | DC/Tumor Fusion Vaccine | Renal cell cancer | 1 | Active, not recruiting | GM-CSF | NCT00458536 | Beth Israel Deaconess Medical Center | USA |
| Hybrid cell membrane vaccine | DC/RCC fusion vaccine | Renal cell cancer | 2 | Terminated(Funding) | Anti-PD1 | NCT01441765 | Beth Israel Deaconess Medical Center | USA |
