# Supplementary figures and images for: Membrane-based cancer nanovaccines: the time is now
Source: QJM. 2023 May 17;116(8):621–4. doi: 10.1093/qjmed/hcad089 (PMC10497184; doi:10.1093/qjmed/hcad089)

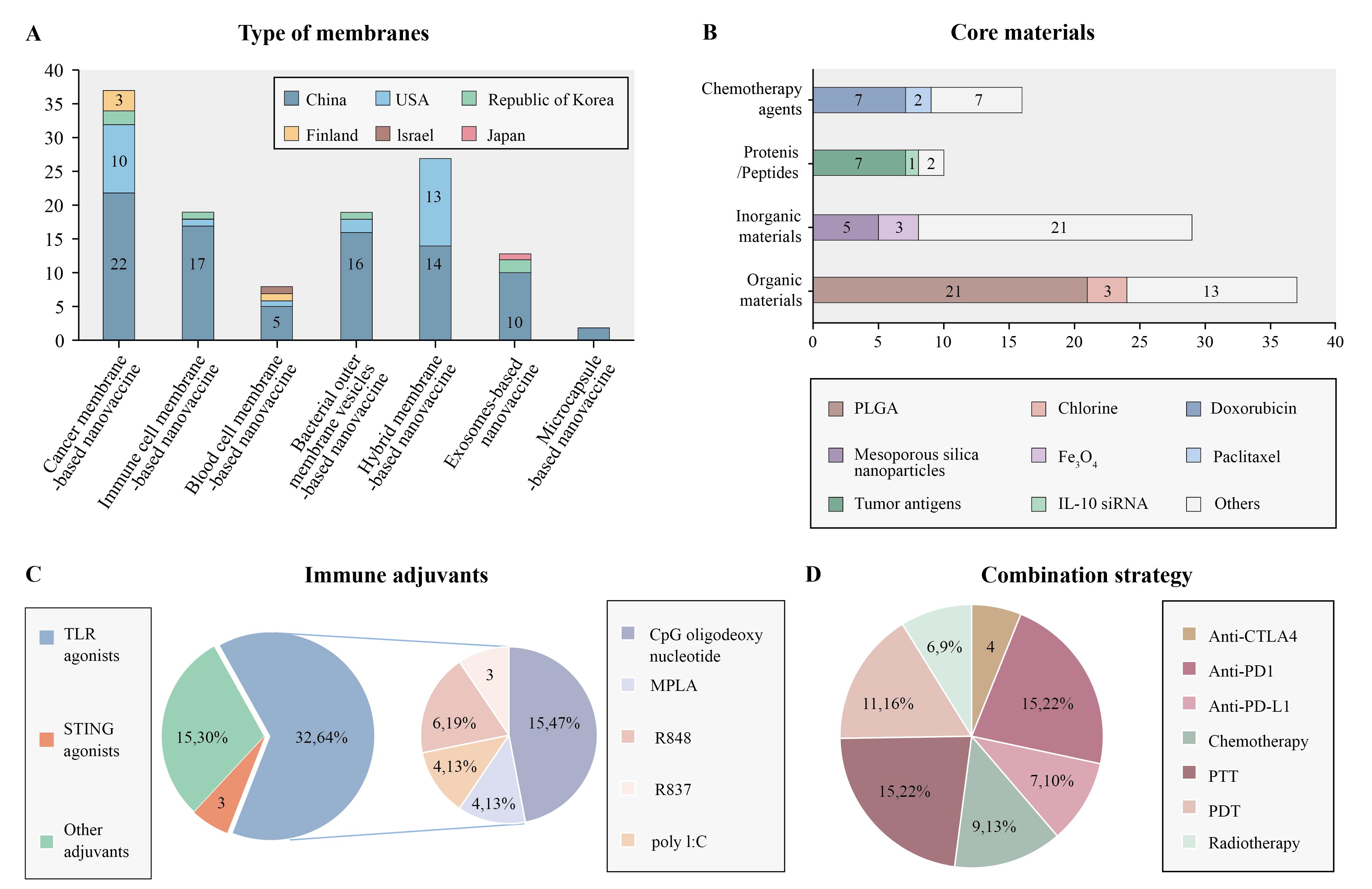

Supplement: hcad089_Supplementary_Data [file hcad089_supplementary_data.zip › hcad089_Supplementary_Data/Figure s1.jpg]
